# Infectious Disease Surveillance by Medical Examiners and Coroners

**DOI:** 10.3201/eid1905.121661

**Published:** 2013-05

**Authors:** Dianna M. Blau, Steven C. Clark, Kurt B. Nolte, the National Association of Medical Examiners Ad-hoc Committee for Bioterrorism and Infectious Diseases

**Affiliations:** Centers for Disease Control and Prevention, Atlanta, Georgia, USA (D.M. Blau);; Occupational Research and Assessment, Big Rapids, Michigan, USA (S.C. Clark);; New Mexico Office of the Medical Investigator, Albuquerque, New Mexico, USA (K.B. Nolte)

**Keywords:** autopsy, infectious disease, surveillance, medical examiners, coroners

**To the Editor:** Medical examiners and coroners (ME/C) investigate ≈20% of all deaths in the United States (*1*); these include persons who die outside the health care system or die precipitously without a confirmed diagnosis. Surveillance through ME/C offices for unexplained deaths that might have infectious causes can serve as a sentinel system to identify new agents, identify notifiable diseases missed by traditional surveillance systems, recognize unique signs and symptoms of known pathogens, and detect bioterrorism (*1*). This surveillance model, called Med-X, is based on standards for autopsy performance, diagnostic testing, and public health reporting and is currently being performed locally in a small number of offices. 

To assess more widely the capacity of ME/C offices to conduct infectious disease surveillance, the National Association of Medical Examiners distributed an Internet-based questionnaire to 155 ME/C offices in the United States that serve populations >300,000; the questionnaires were completed during August–September 2009. Survey questions addressed interest in and physical, personnel, and logistical capacities for conducting surveillance for deaths that could have resulted from infectious diseases. Because many infections can be transmitted during autopsy, specific biosafety features for the autopsy suite were also assessed.

The ME/C offices that responded (68/155) are responsible for 59% of the population served by the target ME/C offices and, on average, perform autopsies on 33% (range 12%–80%) of their cases. Most of the responding offices were the principal office for the area, which was primarily at the county or parish level. Of the responding offices, 97% indicated an interest in a medical examiner–based surveillance system for infectious diseases; 13% currently identify and report cases through the Med-X system. Almost half of the respondents noted some Biosafety Level 3 features in their facilities, including negative pressure ventilation, double-door entry into autopsy suites, or appropriate air exchange and ventilation systems. With respect to current capabilities and practices of surveillance of infectious diseases, most respondents had optimal databases that contained complete and searchable data that included circumstances of death narrative, autopsy findings, and laboratory results. Most offices also had established practices of identifying infectious diseases and of reporting to local or state health departments notifiable and nonnotifiable diseases. 

The most often cited barriers to participation in ME/C infectious disease surveillance were funding and resources (85%), lack of supplies (76%), insufficient laboratory testing capability (69%), and personnel requirements (63%). These factors all relate primarily to the subsequent autopsies resulting from the surveillance. With respect to current autopsy practices, survey results suggest that inadequate usage of personal protective equipment (6%), lack of autopsy suites with negative pressure (21%), and inadequate required vaccinations (e.g., hepatitis B) for pathologists (40%) are areas where improvement is needed.

During the past few decades, several diseases of public health importance, including new or emerging infectious diseases, have been recognized and identified through the collaborative efforts of public health partners and medical examiners, performance of autopsies, and subsequent postmortem diagnostic testing (*2*–*4*). The findings from this survey suggest that interest and potential exist for the establishment of an enhanced national ME/C-based surveillance system for novel or emerging infectious diseases and bioterrorism. A surveillance protocol is already available for distribution (*5*). Although survey respondents showed high interest in such a system, this result may be an overestimation because of the offices targeted and the low overall response rate. Addressing existing barriers, including funding and infrastructure deficiencies, may increase participation in such a national surveillance system. Development of a national surveillance system of this type would require fulfilling recently identified steps needed to strengthen the competency of national death investigation systems (*6*), establishment of uniform statewide and interstate standards of operation such as those outlined in the National Association of Medical Examiners accreditation checklist (*7*), consolidation of smaller offices, regionalization of services, and standardization of staff training.
